# Assessment of existing anthropometric indices for screening sarcopenic obesity in older adults

**DOI:** 10.1017/S0007114522001817

**Published:** 2023-03-14

**Authors:** Jin Eui Kim, Jimi Choi, Miji Kim, Chang Won Won

**Affiliations:** 1 Department of Biomedical Science and Technology, Graduate School, Kyung Hee University, Seoul 02447, Republic of Korea; 2 Division of Endocrinology and Metabolism, Department of Internal Medicine, College of Medicine, Korea University, Seoul 02841, Republic of Korea; 3 Department of Biomedical Science and Technology, College of Medicine, East-West Medical Research Institute, Kyung Hee University, Seoul 02447, Republic of Korea; 4 Department of Family Medicine, College of Medicine, Kyung Hee University, Seoul 02447, Republic of Korea

**Keywords:** Anthropometry, Sarcopenia, Obesity, Ageing, Body composition

## Abstract

Sarcopenic obesity is defined as the presence of high fat mass and low muscle mass combined with low physical function, and it is closely related with the onset of cardiovasular diseases (CVD). The existing anthropometric indices, which are being utilised in clinical practice as predictors of CVD, may also be used to screen sarcopenic obesity, but their feasibility remained unknown. Using cross-sectional data of 2031 participants aged 70–84 years (mean age, 75·9 ± 3·9 years; 49·2 % women) from the Korean Frailty and Aging Cohort Study, we analysed the association of anthropometric indices, including body mass index (BMI), waist circumference (WC), waist-to-height ratio (WHtR) and weight-adjusted waist index (WWI) with sarcopenic obesity. Body composition was measured using dual-energy X-ray absorptiometry. Higher WWI, WHtR and WC quartiles were associated with higher risk of sarcopenic obesity; the odds ratio (OR) of sarcopenic obesity were highest in the fourth quartile of the WWI (OR: 10·99, 95 % CI: 4·92–24·85, *P*_for trend_ < 0·001). WWI provided the best diagnostic power for sarcopenic obesity in men (area under the receiver operating characteristic curve: 0·781, 95 % CI: 0·751–0·837). No anthropometric indices were significantly associated with sarcopenic obesity in women. WWI was the only index that was negatively correlated with physical function in both men and women. WWI showed the strongest association with sarcopenic obesity, defined by high fat mass and low muscle mass combined with low physical function only in older men. No anthropometric indices were associated with sarcopenic obesity in older women.

Sarcopenic obesity is the coexistence of sarcopenia and obesity, which is characterised by age-related changes in body composition, decreased muscle mass, increased fat mass and decreased muscle strength and physical performance^(1)^. It is a common problem in older adults that results in physical disability as well as increased cardiovascular diseases (CVD) morbidity and mortality^(2–4)^. While earlier diagnosis and treatment is imperative, sarcopenic obesity lacks a standardised diagnostic criterion and has traditionally been defined as low muscle mass and high-fat mass^(5,6)^. Dual-energy X-ray absorptiometry (DXA), MRI and CT are the most precise and accurate methods for measuring body composition; however, these methods have several limitations in clinical practice. They are expensive and time consuming; furthermore, CT is potentially hazardous because of radiation exposure^(7,8)^. More importantly, such equipment lacks portability, which makes it difficult to utilise them in extramural care settings. Therefore, it is crucial to develop a method to diagnose sarcopenic obesity in diverse settings while keeping in mind factors such as cost-effectiveness, time taken and safety.

Anthropometry is a simple and practical method to estimate body composition^(9)^, and there are several anthropometric indices that have been used widely in epidemiologic studies. The global standard to define obesity is based on body mass index (BMI); waist circumference (WC) and waist-to-height ratio (WHtR) were developed for indicators of abdominal obesity. Those anthropometric indices are strongly associated with the onset of CVD^(10–13)^, and CVD is significantly associated with sarcopenic obesity. This may imply the possibility of anthropometric indices for screening sarcopenic obesity. However, BMI has shown a J-shaped relationship with CVD mortality, which resulted in the obesity paradox^(14–16)^, possibly because of its inability to discriminate muscle mass from fat mass^(17,18)^. In other words, BMI cannot reflect low muscle mass and high fat mass simultaneously. After the studies revealed that abdominal fat, especially visceral fat, is strong predictor of all-cause and CVD mortality^(19,20)^, WC and WHtR were proposed in order to overcome the limitation BMI. WC and WHtR had shown a significantly higher association with CVD mortality compared with BMI; however, similar obesity paradox phenomenon was observed^(21)^. In addition, a recent study suggested a high correlation among WC, WHtR and BMI^(22)^, which represents that high WC and WHtR may also be due to high muscle mass, not solely by high-fat mass. Therefore, the adequacy of anthropometric indices as independent indicators of sarcopenic obesity is still not clear and has to be tested before clinical application. At the same time, a development of new anthropometric indices that can reciprocally reflect muscle mass and fat mass is needed.

In 2018, a new anthropometric index called the weight-adjusted waist index (WWI), which standardised WC for weight, was developed to overcome the shortcomings of existing anthropometric indices^(23)^. The study showed that WWI had a relatively consistent and linear relationship with both CVD morbidity and mortality. More recently, WWI was shown to discriminate muscle mass from fat mass as it showed a negative association with muscle mass and a positive association with fat mass in older adults^(24)^, suggesting WWI as a possible indicator of sarcopenic obesity. Along with the change in muscle mass, other components that are proposed to diagnose sarcopenia are muscle strength and physical performance^(25–28)^; however, not only the association of WWI with muscle strength and physical performance has not yet been determined but also the studies regarding existing anthropometric indices and physical function are limited^(29,30)^.

In this study, we aimed to analyse the association of different anthropometric indices, including BMI, WC, WHtR and WWI, with sarcopenic obesity to compare their feasibility for screening sarcopenic obesity in community-dwelling older adults.

## Methods

### Study population

The Korean Frailty and Aging Cohort Study (KFACS) is a nationwide multicentre cohort study that was primarily designed to assess the frailty status of community-dwelling older adults in South Korea. The participants were sex- and age-stratified community residents recruited from urban and rural areas around ten centres who were ambulatory with or without walking aids. The age ratio was 6:5:4 for age 70–74, 75–79 and 80–84 years, respectively, and the sex ratio was 1:1. Followed by the suggestion from the frailty consensus^(31)^, the starting age of the KFACS was set from 70. The participants over 85 years were excluded due to their difficulty of centre visits and follow-up surveys; the advanced age over 85 years also had a higher probability of interrupting the identification of physical frailty-associated risk factors. Overall, the inclusion criteria of the participant were age 70–84 years, currently living in the community, having no problem with communication and no prior diagnosis of dementia. The baseline study comprised face-to-face interviews, health examinations and laboratory tests with a total of 3013 participants. Among the total participants, 2403 underwent body composition measurement with DXA in eight university hospitals and 610 with bioelectrical impedance analysis in two community centres. For this study, those who underwent bioelectrical impedance analysis were excluded because of possible systematic bias between DXA and bioelectrical impedance analysis^(32)^. The final analysis included 2031 participants after excluding 321 participants who had artificial joints, pins, plates or other types of metal objects in any part of their bodies, as metal implants could have affected the measurement accuracy of appendicular skeletal muscle mass (ASM) or percentage of body fat^(33)^, and fifty-one participants who had missing data for the diagnostic criteria of sarcopenic obesity. The details of the KFACS protocol have been described previously^(34)^.

### Ethics

The KFACS protocol was conducted according to the guidelines laid down in the Declaration of Helsinki, and all procedures involving human subjects/patients were approved by the Institutional Review Board of the Clinical Research Ethics Committee of the Kyung Hee University Medical Center. Written informed consent was obtained from all participants (IRB number: 2015-12-103). This study was approved as an exempt from the Institutional Review Board review (IRB number: 2021-02-021).

### Anthropometric measurements

The height, weight and WC of all the participants were recorded. The height and WC were measured to the nearest 0·1 cm, and weight was measured to the nearest 0·1 kg. BMI was calculated as weight (kg) divided by the square of the height (m^2^). WC (cm) was measured at the midpoint between the lower end of the last rib and the upper ridge of the iliac crest. WHtR was calculated as WC (cm) divided by height (cm)^(35)^, and WWI was calculated as WC (cm) divided by the square root of the weight (√kg). Details regarding the derivation of WWI have been described in a previous study^(23)^.

### Body composition measurements

ASM and percentage of body fat were measured using DXA (Lunar, GE Healthcare, Madison, WI; Hologic DXA, Hologic Inc.). The participants were asked to remove all metal accessories before the scan and lie in a supine position on the scanner table with their limbs placed parallel to their bodies, according to the manufacturer’s protocol. ASM was calculated as the sum of the lean masses of the right and left arms and legs. ASM index was defined as ASM/height^2^ (kg/m^2^)^(6)^. Our laboratory assessment of forty volunteers with repositioning between scans demonstrated that the coefficients of variation for whole-body composition were < 2·5 %.

### Physical function measurements

#### Muscle strength

Muscle strength was evaluated by grip strength and measured using a digital handgrip dynamometer (T.K.K.5401; Takei Scientific Instruments Co. Ltd). The participants were asked to stand upright, place their shoulder in a neutral position with both arms fully extended and hold the dynamometer for 3 s with maximum strength. The strength was measured twice for each hand at 3-min intervals. The best records for each hand were rounded to the nearest 0·1 kg^(36)^.

#### Physical performance

Physical performance was evaluated by 4-m usual gait speed, the five-times sit-to-stand test and the Short Physical Performance Battery (SPPB). The 4-m usual gait speed was measured using an automatic gait speed meter (Dynamicphysiology), with acceleration and deceleration phases of 1·5 m each^(37)^. The participants performed two trials with their usual walking paces, and the average rounded to the nearest 0·01 m/s was taken for the analysis. The five-times sit-to-stand test was conducted by measuring the time it took for the participants to stand five times from a sitting position as quickly as possible from a straight-backed armchair without using their arms^(38)^. The SPPB consists of the 4-m usual gait speed test measures, five-times sit-to-stand test measures and three standing balance measures^(38)^. In the standing balance test, the participants were first asked to stand with their feet placed together as close as possible, then in a semi-tandem position, and finally in a tandem position for 10 s. Each item of the SPPB was scored on a scale of 0–4 based on the normative scores obtained from the Established Population for Epidemiologic Studies of the Elderly, which makes the total possible score between 0 and 12^(39)^.

#### Definitions of sarcopenic obesity

While there is no global consensus to define obesity by the percentage of body fat^(40)^, the commonly used definition of obesity for older population was suggested in the previous research of New Mexico Aging Process Study. It defined obesity as the percentage of body fat greater than 60th percentile in the study population resulting in cut-off values of ≥ 28 % for men and ≥40 % for women^(5,41)^. Followed by the standard of the New Mexico Aging Process Study, we defined obesity as a high total fat mass greater than 60th percentile of our study population according to the percentage of body fat. The resulting cut-off values were ≥ 28·2 % for men and ≥ 38·8 % for women, which correspond to those of the New Mexico Aging Process Study.

Three different definitions of sarcopenia were established based on the Asian Working Group for Sarcopenia 2019 consensus^(25)^, which were as follows: (1) low muscle mass, (2) low muscle mass with low muscle strength and/or slow gait speed and (3) low muscle mass with low muscle strength and/or physical performance (slow gait speed, poor performance in the five-times sit-to-stand test and/or low SPPB score). By combining the definitions of obesity and sarcopenia, we established the following three sets of diagnostic criteria for sarcopenic obesity:

Criterion 1: High fat mass + low muscle mass

Criterion 2: High fat mass + low muscle mass + low muscle strength and/or slow gait speed

Criterion 3: High fat mass + low muscle mass + low muscle strength and/or physical performance

Low muscle mass was defined as an ASM/height^2^ value of < 7·00 kg/m^2^ for men and < 5·40 kg/m^2^ for women; low muscle strength was defined as a grip strength of < 28 kg for men and < 18 kg for women; the cut-off scores for the 4-m usual gait speed, five-times sit-to-stand test and SPPB for low physical performance were < 1·0 m/s, ≥ 12 s and ≤ 9, respectively, for both sexes^(25)^.

### Statistical analyses

Data are presented as mean ± standard deviation (sd) for continuous variables and as numbers (percentages) for categorical variables. Continuous variables with skewed distributions are reported as median (interquartile range). To assess the differences in characteristics between the sexes, the means or medians of the two groups were compared using the Student’s *t* test or Mann–Whitney *U*-test, respectively. The percentages of categorical variables were compared using the *χ* ^2^ or Fisher’s exact test, as appropriate. As there was a significant sex-specific difference in the association between the anthropometric indices and sarcopenic obesity in the exploratory data analysis, all the analyses were stratified by sex. The association between the anthropometric indices and sarcopenic obesity was evaluated in the unadjusted and age-adjusted model using binary logistic regression. The results were reported as odds ratio (OR) according to the quartiles of each anthropometric index and corresponding 95 % CI to compare the strengths of the associations of the indices measured on different scales. The OR per sd were calculated using a multiple logistic regression model, and the predicted probability calculated from this model was used to evaluate the discriminative ability of the indices for sarcopenic obesity by analysing the receiver operating characteristic curve and the area under the receiver operating characteristic curve (AUC) (95 % CI). The AUC of the anthropometric indices were compared using DeLong’s method^(42)^. The correlation between the anthropometric indices and continuous components of sarcopenic obesity was evaluated using Pearson’s or Spearman’s correlation analysis, according to the distribution of variables. Statistical significance was set at a *P* < 0·05. All statistical analyses were performed using SAS software (version 9.4; SAS Institute Inc.) and R (version 4.0.3; R Foundation for Statistical Computing, Vienna, Austria). This study is a secondary analysis; the sample size was determined by recruitment in the KFACS^(34)^ and satisfied the rule of ten events per variable in logistic regression analysis^(43)^.

## Results

The characteristics of the study participants are listed in Table 1. This study included 1032 men and 999 women. Men were significantly older (76·4 ± 3·9 years *v*. 75·4 ± 3·9 years), had a higher WC (88·4 ± 8·3 cm *v*. 86·0 ± 8·2 cm), a lower BMI (23·9 ± 2·8 *v*. 24·4 ± 2·8), WHtR (0·54 ± 0·05 cm *v*. 0·57 ± 0·05 cm) and WWI (11·0 ± 0·6 cm/√kg *v*. 11·5 ± 0·7 cm/√kg) compared with women. Men were also more likely to have higher incidence rates of myocardial infarction, cerebrovascular disease, diabetes mellitus and chronic obstructive pulmonary disease than women. On the other hand, women were more likely to have higher incidence rates of hypertension, dyslipidaemia, osteoarthritis, rheumatoid arthritis, osteoporosis and asthma than men. The prevalence of sarcopenic obesity was higher in men than in women according to criteria 1 (21·8 % *v*. 14·3 %), 2 (10·5 % *v*. 7·0 %) and 3 (12·7 % *v*. 10·3 %); the difference between men and women was not significant in criteria 3 (*P* = 0·093). The percentage of body fat was lower in men than in women (26·3 ± 6·0 % *v*. 36·8 ± 5·9 %). Although men had higher ASM (19·2 ± 2·7 kg *v*. 13·5 ± 1·8 kg) and ASM/height^2^ (7·0 ± 0·8 kg/m^2^ *v*. 5·8 ± 0·7 kg/m^2^) than women, the proportion of low muscle mass was also higher in men than in women (48·3 % *v*. 27·1 %). The difference between the sexes in the proportion of low muscle strength was not significant (21·7 % in men and 19·0 % in women, *P* = 0·133). The proportion of slow gait speed (23·3 % *v*. 33·1 %) and low physical performance (39·5 % *v*. 53·9 %) was lower in men than in women.

**Table 1.** Characteristics of the study participants (Mean values and standard deviations)

	Total		Men		Women		
	Mean		Mean		Mean		
Variables	(*n* 2031)	sd	(*n* 1032)	sd	(*n* 999)	sd	*P*
Age, years	75·9	3·9	76·4	3·9	75·4	3·9	< 0·001
Height, cm	158·5	8·4	164·9	5·7	151·9	5·2	< 0·001
Weight, kg	60·8	9·3	65·0	8·9	56·5	7·7	< 0·001
BMI, kg/m^2^	24·1	2·8	23·9	2·8	24·4	2·8	< 0·001
WC, cm	87·2	8·3	88·4	8·3	86·0	8·2	< 0·001
WHtR, cm	0·55	0·05	0·54	0·05	0·57	0·05	< 0·001
WWI, cm/√kg	11·2	0·7	11·0	0·6	11·5	0·7	< 0·001
Percentage of body fat	31·5	7·9	26·3	6·0	36·8	5·9	< 0·001
SBP, mmHg	131·1	15·6	131·1	15·3	131·1	15·9	0·962
DBP, mmHg	77·4	9·1	78·0	9·1	76·9	9·2	0·009

	*n*	%	*n*	%	*n*	%	
Comorbidity							
Hypertension	1122	55·3	546	53·0	576	57·7	0·033
Dyslipidaemia	654	32·6	249	24·4	405	41·2	< 0·001
Myocardial infarction	51	2·5	39	3·8	12	1·2	< 0·001
Heart failure	11	0·6	6	0·6	5	0·5	0·822
Angina	113	5·6	62	6·0	51	5·2	0·399
Peripheral artery disease	17	0·8	11	1·1	6	0·6	0·258
CVD	89	4·4	60	5·8	29	2·9	0·001
Diabetes mellitus	432	21·3	244	23·7	188	18·9	0·008
Osteoarthritis	384	19·0	106	10·3	278	28·0	< 0·001
Rheumatoid arthritis	38	1·9	8	0·8	30	3·0	< 0·001
Osteoporosis	259	12·9	26	2·5	233	23·8	< 0·001
Asthma	65	3·2	25	2·4	40	4·0	0·043
COPD	23	1·1	20	1·9	3	0·3	0·001

	Mean	sd	Mean	sd	Mean	sd	
Laboratory results							
Creatinine, mg/dl	0·85	0·28	0·98	0·30	0·72	0·16	< 0·001
eGFR, ml/min/1·73 m^2^	81·2	19·0	79·0	19·3	83·4	18·6	< 0·001
FBS							
Median	97		99		96		< 0·001
IQR	90–110		91–112		89–108		
Total cholesterol, mg/dl	175·2	35·7	168·6	34·7	182·1	35·5	< 0·001
Triglyceride							
Median	105		100		110		< 0·001
IQR	79–142		75–137		83–146		
HDL-cholesterol, mg/dl	53·0	14·2	50·8	14·3	55·2	13·8	< 0·001
LDL-cholesterol, mg/dl	108·9	33·0	104·5	31·7	113·4	33·9	< 0·001
HbA1c, %	6·0	0·8	6·0	0·8	6·0	0·8	0·158
Skeletal muscle mass							
ASM by DXA, kg	16·4	3·7	19·2	2·7	13·5	1·8	< 0·001
ASM/height^2^, kg/m^2^	6·4	1·0	7·0	0·8	5·8	0·7	< 0·001
Low muscle mass							
*n*	769		498		271		< 0·001
%	37·9		48·3		27·1		
Maximum grip strength, kg	27·0	7·4	32·5	5·7	21·3	3·9	< 0·001
Low muscle strength							
*n*	414		224		190		0·133
%	20·4		21·7		19·0		
Physical performance							
4-m usual gait speed, m/s	1·1	0·3	1·2	0·3	1·1	0·2	< 0·001

	*n*	%	*n*	%	*n*	%	
4-m usual gait speed < 1·0 m/s	571	28·1	240	23·3	331	33·1	< 0·001
Five-times sit-to-stand test							
Median	10·5		10·0		11·2		< 0·001
IQR	8·7–12·9		8·4–12·3		9·2–13·7		
Five-times sit-to-stand test ≥ 12 s	688	33·9	287	27·8	401	40·1	< 0·001
SPPB score, median (IQR)							
Median	11		12		11		< 0·001
IQR	10–12		11–12		10–12		
SPPB score ≤ 9 points	294	14·5	102	9·9	192	19·2	< 0·001
Low physical performance	946	46·6	408	39·5	538	53·9	< 0·001
Sarcopenic obesity							
Criterion 1	368	18·1	225	21·8	143	14·3	< 0·001
Criterion 2	178	8·8	108	10·5	70	7·0	0·006
Criterion 3	234	11·5	131	12·7	103	10·3	0·093

WC, waist circumference; WHtR, waist-to-height ratio; WWI, weight-adjusted waist index; SBP, systolic blood pressure; DBP, diastolic blood pressure; CVD, cardiovascular diseases; COPD, chronic obstructive pulmonary disease; eGFR, estimated glomerular filtration rate; FBS, fetal bovine serum; ASM, appendicular skeletal muscle mass; DXA, dual-energy X-ray absorptiometry; SPPB, short physical performance battery; IQR, interquartile range.

Criterion 1: high-fat mass + low muscle mass; Criterion 2: high-fat mass + low muscle mass + low muscle strength and/or slow gait speed and Criterion 3: high-fat mass + low muscle mass + low muscle strength and/or low physical performance.

High-fat mass: body fat percentage of ≥ 28·2 % for men and ≥ 38·8 % for women; low muscle mass: ASM/height^2^ of < 7·00 kg/m^2^ for men and < 5·40 kg/m^2^ for women; low muscle strength: grip strength of < 28 kg for men and < 18 kg for women; slow gait speed: 4-m usual gait speed of < 1·0 m/s and low physical performance: five-times sit-to-stand test score of ≥ 12 s, 4-m usual gait speed of < 1·0 m/s and/or SPPB score of ≤ 9.

Variables are expressed as means ± standard deviation for continuous variables and as *n* (%) for categorical variables. Continuous variables with skewed distributions were reported as median (IQR). *P* values were obtained using the *χ* ^2^ test, Fisher’s exact test or Student’s *t* test, as appropriate.

### Association between anthropometric indices and sarcopenic obesity

**Fig. 1.**
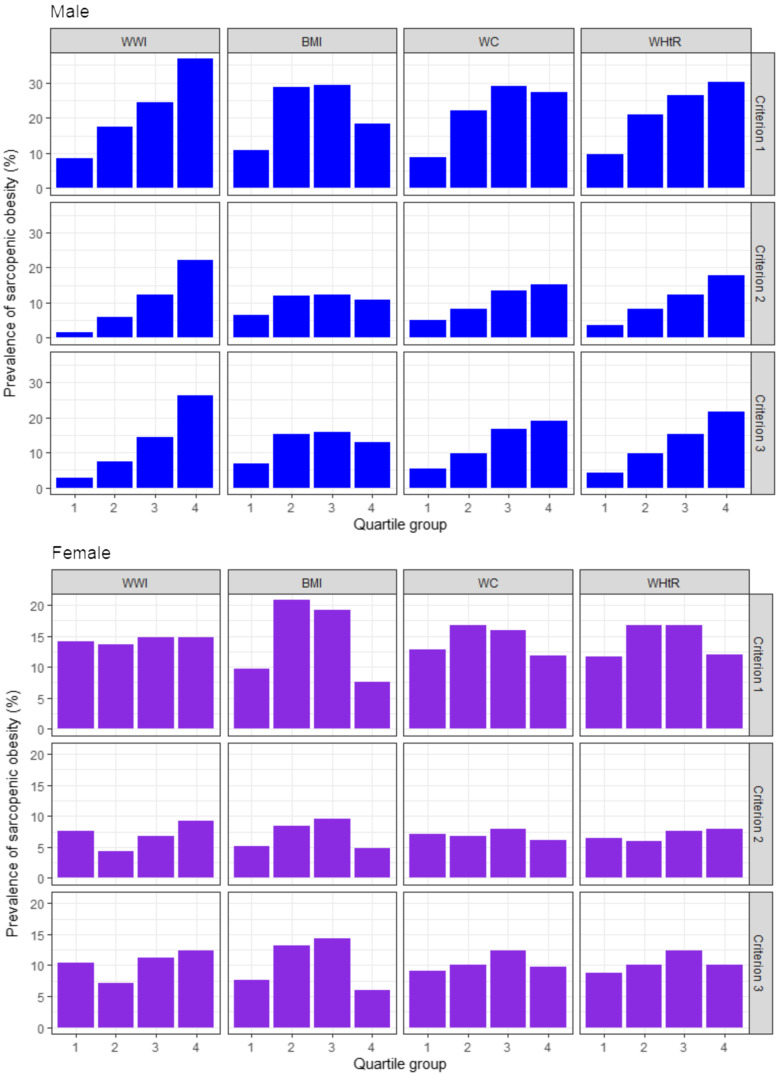
Age-adjusted prevalence of sarcopenic obesity according to the quartiles of the anthropometric indices. WWI, weight-adjusted waist index; WC, waist circumference; WHtR, waist-to-height ratio. Criterion 1: high fat mass + low muscle mass; Criterion 2: high fat mass + low muscle mass + low muscle strength and/or slow gait speed; Criterion 3: high fat mass + low muscle mass + low muscle strength and/or low physical performance. High fat mass: body fat percentage of ≥ 28·2 % for men and ≥ 38·8 % for women; low muscle mass: appendicular skeletal muscle mass/height^2^ of < 7·00 kg/m^2^ for men and < 5·40 kg/m^2^ for women; low muscle strength: grip strength of < 28 kg for men and < 18 kg for women; slow gait speed: 4-m usual gait speed of < 1·0 m/s and low physical performance: five-times sit-to-stand test score of ≥ 12 s, 4-m usual gait speed of < 1·0 m/s and/or short physical performance battery score of ≤ 9.

The age-adjusted prevalence of sarcopenic obesity was high in men with higher WWI and WHtR for all the three diagnostic criteria, but not in women with higher WWI and WHtR (Fig. 1). Followed by the result, we analysed the age-adjusted OR of sarcopenic obesity according to the quartiles of the anthropometric indices as illustrated in Table 2. In men, higher WWI and WHtR quartiles were associated with higher risk of sarcopenic obesity for all the three diagnostic criteria, with the OR being the highest in the fourth quartiles of the WWI (OR: 5·84, 95 % CI: 3·51, 9·70, *P*_for trend_ < 0·001 in criterion 1; OR: 14·64, 95 % CI: 5·20, 41·25 in criterion 2, *P*_for trend_ < 0·001; and OR: 10·99, 95 % CI: 4·92, 24·85, *P*_for trend_ < 0·001 in criterion 3) and WHtR (OR: 3·94, 95 % CI: 2·41, 6·45, *P*_for trend_ < 0·001 in criterion 1; OR: 5·73, 95 % CI: 2·72–12·05, *P*_for trend_ < 0·001 in criterion 2; and OR: 5·99, 95 % CI: 3·05, 11·79, *P*_for trend_ < 0·001 in criterion 3). The OR of sarcopenic obesity were higher in the fourth quartiles of the WWI than in those of the WHtR, especially based on criteria 2 and 3, which included muscle strength and/or physical performance as diagnostic components of sarcopenic obesity. WC also showed the highest OR in the fourth quartiles on criteria 2 (OR: 3·61, 95 % CI: 1·86, 7·01, *P*_for trend_ < 0·001) and 3 (OR: 4·32, 95 % CI: 2·31, 8·10, *P*_for trend_ < 0·001), but their values were lower compared with WWI and WHtR. Meanwhile, no anthropometric indices were significantly associated with sarcopenic obesity in women. Similar results were observed in the unadjusted model in both men and women (online Supplementary Table S1).

**Table 2.** Age-adjusted odds ratios of sarcopenic obesity according to the quartiles of the anthropometric indices (Odd ratio and 95 % confidence intervals)

		Criterion 1				*P*	Criterion 2				*P*	Criterion 3				*P*
Quartile group	Range	*n*	%	Age-adjusted OR	95 % CI		*n*	%	Age-adjusted OR	95 % CI		*n*	%	Age-adjusted OR	95 % CI	
WWI																
Men																
1st	9·1–10·6	22	8·5	1 (Reference)			4	1·6	1 (Reference)			7	2·7	1 (Reference)		
2nd	10·6–11·0	45	17·4	2·25	1·31, 3·88	0·003	15	5·8	3·83	1·25, 11·76	0·019	19	7·4	2·80	1·16, 6·81	0·023
3rd	11·0–11·4	63	24·4	3·34	1·98, 5·63	< 0·001	32	12·4	8·10	2·81, 23·35	< 0·001	37	14·3	5·53	2·41, 12·70	< 0·001
4th	11·4–12·9	95	36·8	5·84	3·51, 9·70	< 0·001	57	22·1	14·64	5·20, 41·25	< 0·001	68	26·4	10·99	4·92, 24·85	< 0·001
*P*_for trend_						< 0·001					< 0·001					< 0·001
Women																
1st	9·5–10·9	35	14·1	1 (Reference)			19	7·6	1 (Reference)			26	10·4	1 (Reference)		
2nd	10·6–11·5	34	13·6	0·95	0·57, 1·57)	0·830	11	4·4	0·53	0·24, 1·14	0·103	18	7·2	0·64	0·34, 1·20	0·163
3rd	11·5–11·9	37	14·8	1·03	0·62, 1·69	0·922	17	6·8	0·78	0·39, 1·55	0·482	28	11·2	0·99	0·56, 1·76	0·980
4th	11·9–14·0	37	14·8	0·95	0·57, 1·58	0·849	23	9·2	0·86	0·45, 1·65	0·656	31	12·4	0·94	0·53, 1·65	0·820
*P*_for trend_						0·938					0·955					0·811
*P*_for interaction_						< 0·001					< 0·001					< 0·001
BMI																
Men																
1st	14·5–22·0	28	10·9	1 (Reference)			17	6·6	1 (Reference)			18	7·0	1 (Reference)		
2nd	22·0–23·9	74	28·7	3·49	2·16, 5·63	< 0·001	31	12·0	2·17	1·16, 4·07	0·016	39	15·1	2·61	1·44, 4·73	0·002
3rd	23·9–25·7	76	29·5	3·61	2·24, 5·83	< 0·001	32	12·4	2·26	1·21, 4·22	0·011	41	15·9	2·77	1·54, 5·01	< 0·001
4th	25·7–33·1	47	18·2	1·94	1·17, 3·21	0·011	28	10·9	1·99	1·05, 3·77	0·035	33	12·8	2·19	1·19, 4·03	0·012
*P*_for trend_						0·029					0·050					0·021
Women																
1st	16·0–22·6	24	9·6	1 (Reference)			13	5·2	1 (Reference)			19	7·6	1 (Reference)		
2nd	22·6–24·4	52	20·8	2·56	1·52, 4·32	< 0·001	21	8·4	1·87	0·90, 3·87	0·091	33	13·2	2·01	1·10, 3·67	0·023
3rd	24·4–26·2	48	19·2	2·33	1·37, 3·94	0·002	24	9·6	2·24	1·10, 4·57	0·026	36	14·4	2·27	1·25, 4·11	0·007
4th	26·2–33·2	19	7·6	0·79	0·42, 1·48	0·456	12	4·8	0·96	0·43, 2·18	0·931	15	6·0	0·80	0·40, 1·63	0·539
*P*_for trend_						0·477					0·877					0·769
*P*_for interaction_						0·166					0·417					0·141
WC																
Men																
1st	60·0–83·0	23	8·9	1 (Reference)			13	5·0	1 (Reference)			14	5·4	1 (Reference)		
2nd	83·1–88·5	57	22·1	2·99	1·77, 5·03	< 0·001	21	8·1	1·82	0·88, 3·75	0·105	25	9·7	1·99	1·01, 3·95	0·048
3rd	88·6–93·9	75	29·1	4·33	2·61, 7·19	< 0·001	35	13·6	3·23	1·65, 6·32	< 0·001	43	16·7	3·73	1·97, 7·05	< 0·001
4th	94·0–115·0	70	27·1	3·91	2·34, 6·51	< 0·001	39	15·1	3·61	1·86, 7·01	< 0·001	49	19·0	4·32	2·31, 8·10	< 0·001
*P*_for trend_						< 0·001					< 0·001					< 0·001
Women																
1st	59·5–80·0	32	12·9	1 (Reference)			18	7·2	1 (Reference)			23	9·2	1 (Reference)		
2nd	80·1–86·0	42	16·8	1·41	0·85, 2·32	0·180	17	6·8	0·99	0·49, 1·99	0·975	25	10·0	1·14	0·63, 2·09	0·665
3rd	86·1–91·5	40	16·0	1·30	0·78, 2·14	0·313	20	8·0	1·14	0·58, 2·23	0·702	31	12·4	1·41	0·79, 2·52	0·239
4th	91·6–111·6	29	11·6	0·88	0·51, 1·51	0·642	15	6·0	0·77	0·38, 1·58	0·478	24	9·6	1·00	0·55, 1·84	0·995
*P*_for trend_						0·605					0·598					0·811
*P*_for interaction_						0·001					0·015					0·007
WHtR																
Men																
1st	0·35–0·51	25	9·7	1 (Reference)			9	3·5	1 (Reference)			11	4·3	1 (Reference)		
2nd	0·51–0·54	54	20·9	2·52	1·51, 4·21	< 0·001	21	8·1	2·59	1·16, 5·82	0·021	25	9·7	2·52	1·21, 5·26	0·014
3rd	0·54–0·57	68	26·4	3·32	2·02, 5·47	< 0·001	32	12·4	3·91	1·81, 8·43	< 0·001	39	15·1	3·99	1·98, 8·01	< 0·001
4th	0·57–0·72	78	30·2	3·94	2·41, 6·45	< 0·001	46	17·8	5·73	2·72, 12·05	< 0·001	56	21·7	5·99	3·05, 11·79	< 0·001
*P*_for trend_						< 0·001					< 0·001					< 0·001
Women																
1st	0·41–0·53	29	11·6	1 (Reference)			16	6·4	1 (Reference)			22	8·8	1 (Reference)		
2nd	0·53–0·57	42	16·8	1·53	0·92, 2·55	0·102	15	6·0	0·94	0·45, 1·96	0·866	25	10·0	1·15	0·63, 2·12	0·647
3rd	0·57–0·60	42	16·8	1·51	0·90, 2·51	0·116	19	7·6	1·15	0·57, 2·32	0·692	31	12·4	1·42	0·79, 2·54	0·239
4th	0·60–0·76	30	12·0	0·95	0·55, 1·64	0·847	20	8·0	1·01	0·51, 2·02	0·974	25	10·0	0·96	0·52, 1·77	0·897
*P*_for trend_						0·823					0·843					0·943
*P*_for interaction_						0·001					0·010					0·001

WWI, weight-adjusted waist index; WC, waist circumference; WHtR, waist-to-height ratio.

Criterion 1: high fat mass + low muscle mass; Criterion 2: high fat mass + low muscle mass + low muscle strength and/or slow gait speed and Criterion 3: high fat mass + low muscle mass + low muscle strength and/or low physical performance.

High fat mass: body fat percentage of ≥ 28·2 % for men and ≥ 38·8 % for women; low muscle mass: appendicular skeletal muscle mass/height^2^ of < 7·00 kg/m^2^ for men and < 5·40 kg/m^2^ for women; low muscle strength: grip strength of < 28 kg for men and < 18 kg for women; slow gait speed: 4-m usual gait speed of < 1·0 m/s and low physical performance: five-times sit-to-stand test score of ≥ 12 s, 4-m usual gait speed of < 1·0 m/s and/or short physical performance battery score of ≤ 9.

*P* values were obtained using a binary logistic regression model.

We also analysed the association of the anthropometric indices with sarcopenic obesity by OR per sd increase (online Supplementary Table S2). The highest OR were identified in WWI in men, whereas no anthropometric indices were significantly associated with sarcopenic obesity in women. We observed an independent association between the anthropometric indices and sarcopenia and obesity (online Supplementary Table S3) and found that WWI was the only index that was positively correlated with sarcopenia in men; thus, the coexistence of sarcopenia and obesity led to an increased association with WWI. However, this association was not observed in women due to the lack of a correlation between WWI and sarcopenia.

### The discriminative ability of the anthropometric indices for predicting sarcopenic obesity

**Fig. 2.**
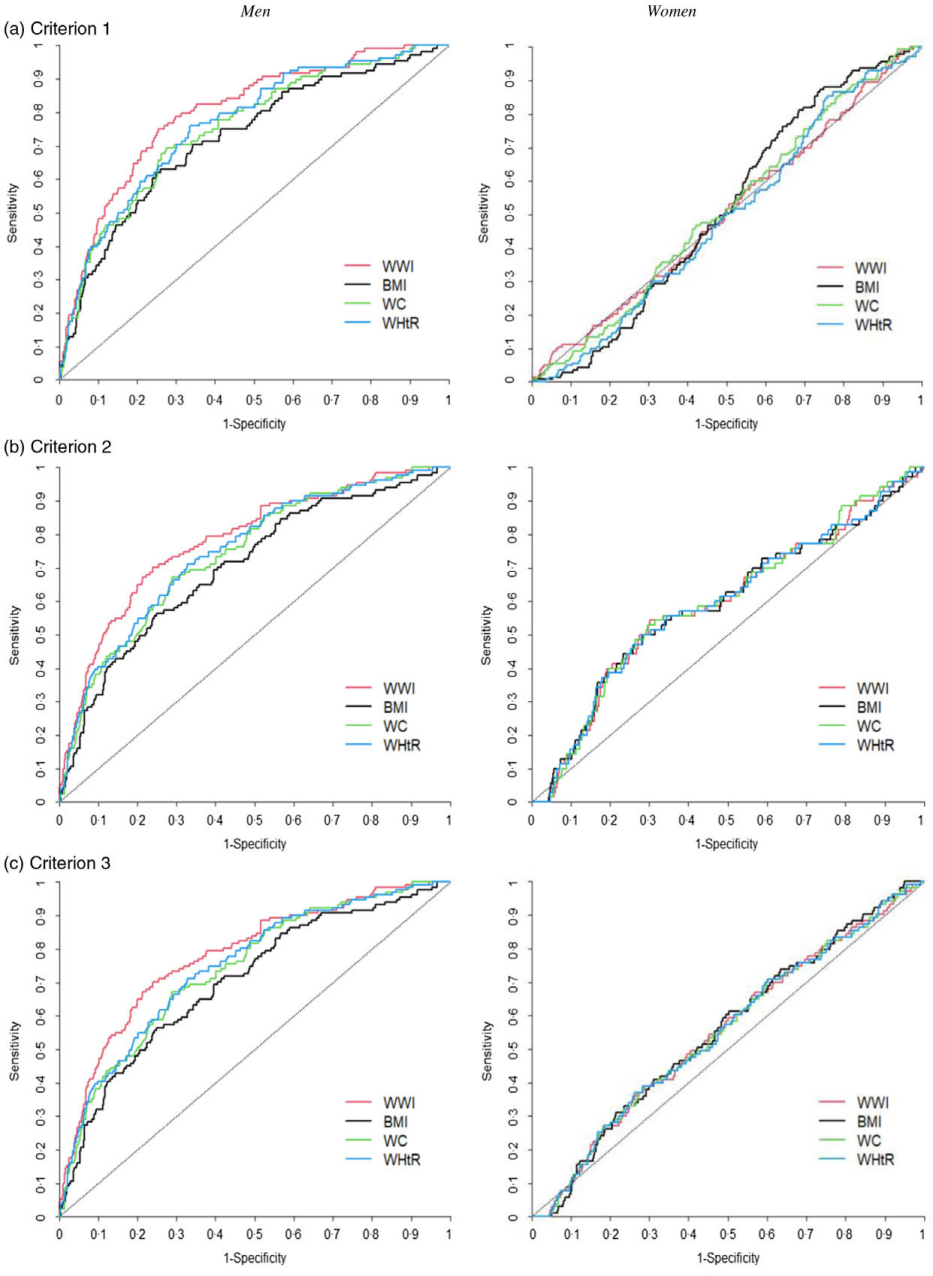
Age-adjusted ROC curves for sarcopenic obesity according to the anthropometric indices. ROC, receiver operating characteristic; WWI, weight-adjusted waist index; WC, waist circumference; WHtR, waist-to-height ratio. Criterion 1: high-fat mass + low muscle mass; Criterion 2: high-fat mass + low muscle mass + low muscle strength and/or slow gait speed; Criterion 3: high-fat mass + low muscle mass + low muscle strength and/or low physical performance. High-fat mass: body fat percentage of ≥ 28·2 % for men and ≥ 38·8 % for women; low muscle mass: appendicular skeletal muscle mass/height^2^ of < 7·00 kg/m^2^ for men and < 5·40 kg/m^2^ for women; low muscle strength: grip strength of < 28 kg for men and < 18 kg for women; slow gait speed: 4-m usual gait speed of < 1·0 m/s and low physical performance: five-times sit-to-stand test score of ≥ 12 s, 4-m usual gait speed of < 1·0 m/s and/or short physical performance battery score of ≤ 9.

The discriminative ability of the anthropometric indices for predicting sarcopenic obesity was determined using the age-adjusted receiver operating characteristic curves for men and women (Fig. 2), and the AUC of the anthropometric indices for the diagnosis of sarcopenic obesity were obtained (online Supplementary Table S4). Although the values were relatively modest, the highest AUC were observed for the WWI (AUC: 0·692, 95 % CI: 0·653, 0·731 in criterion 1; AUC: 0·799, 95 % CI: 0·755, 0·842 in criterion 2 and AUC: 0·781, 95 % CI: 0·738, 0·824 in criterion 3) in men; the WWI had the best diagnostic power for sarcopenic obesity followed by WHtR, WC and BMI according to all the criteria (all *P*_for difference_ in AUC < 0·05). In women, the diagnostic power of anthropometric indices could not be determined because the difference between the AUC of anthropometric indices was not significant according to all the criteria. We obtained similar results for both men and women in the unadjusted model (online Supplementary Fig. S1 and Supplementary Table S5).

The optimal cut-off values, sensitivity and specificity of the anthropometric indices for the diagnosis of sarcopenic obesity in men were also reported, according to Youden’s index (online Supplementary Table S6).

### Correlation between the anthropometric indices and components of sarcopenic obesity

The correlation between the anthropometric indices and each diagnostic component of sarcopenic obesity is shown in Table 3. All anthropometric indices were positively correlated with the percentage of body fat in both men and women. WC showed the strongest correlation (*r* = 0·695, *P* < 0·001) in men followed by WHtR (*r* = 0·689, *P* < 0·001), BMI (*r* = 0·652, *P* < 0·001) and WWI (*r* = 0·480, *P* < 0·001). In women, BMI showed the strongest correlation with the percentage of body fat (*r* = 0·662, *P* < 0·001) followed by WC (*r* = 0·542, *P* < 0·001), WHtR (*r* = 0·499, *P* < 0·001) and WWI (*r* = 0·146, *P* < 0·001). WWI had the lowest correlation coefficient for percentage of body fat in both men and women. However, WWI was the only index that showed a negative correlation with ASM/height^2^ (*r* = –0·073, *P* = 0·020) in men, which confirmed that the WWI discriminated muscle mass from fat mass. Other indices did not discriminate muscle mass from fat mass in men; BMI (*r* = 0·602, *P* < 0·001), WC (*r* = 0·351, *P* < 0·001) and WHtR (*r* = 0·339, *P* < 0·001) all showed positive correlation with ASM/height^2^. In women, all the anthropometric indices did not discriminate muscle mass from fat mass; they all showed positive correlation with ASM/height^2^ (BMI (*r* = 0·483, *P* < 0·001); WC (*r* = 0·348, *P* < 0·001); WHtR (*r* = 0·358, *P* < 0·001) and WWI (*r* = 0·102, *P* = 0·001)).

**Table 3.** Correlation between the anthropometric indices and diagnostic components of sarcopenic obesity (Coefficients and 95 % confidence intervals)

	WWI			BMI			WC			WHtR		
	*r*	95 % CI	*P*	*r*	95 % CI	*P*	*r*	95 % CI	*P*	*r*	95 % CI	*P*
Men *n* 1032												
Percentage of body fat*	0·480	0·431, 0·526	< 0·001	0·652	0·616, 0·686	< 0·001	0·695	0·662, 0·725	< 0·001	0·689	0·656, 0·720	< 0·001
ASM/height^2*^	–0·073	–0·133, –0·012	0·020	0·602	0·562, 0·639	< 0·001	0·351	0·297, 0·404	< 0·001	0·339	0·284, 0·392	< 0·001
Maximum grip strength*	–0·245	–0·302, –0·187	< 0·001	0·182	0·123, 0·241	< 0·001	0·105	0·044, 0·165	0·001	–0·026	–0·086, 0·035	0·411
4-m usual gait speed*	–0·165	–0·224, –0·105	< 0·001	–0·009	–0·070, 0·052	0·773	–0·058	–0·119, 0·003	0·061	–0·101	–0·161, –0·040	0·001
Five-times-sit-to-stand test†	0·178	0·118, 0·236	< 0·001	–0·031	–0·092, 0·030	0·322	0·076	0·015, 0·136	0·015	0·097	0·036, 0·157	0·002
SPPB score†	–0·138	–0·197, –0·077	< 0·001	0·077	0·016, 0·137	0·014	–0·027	–0·088, 0·034	0·392	–0·048	–0·109, 0·013	0·124
Women *n* 999												
Percentage of body fat*	0·146	0·085, 0·206	< 0·001	0·662	0·626, 0·696	< 0·001	0·542	0·497, 0·585	< 0·001	0·499	0·451, 0·544	< 0·001
ASM/height^2*^	0·102	0·041, 0·163	0·001	0·483	0·434, 0·529	< 0·001	0·348	0·292, 0·401	< 0·001	0·358	0·303, 0·411	< 0·001
Maximum grip strength*	–0·214	–0·272, –0·154	< 0·001	0·070	0·008, 0·131	0·028	0·036	–0·027, 0·097	0·262	–0·096	–0·157, –0·034	0·002
4-m usual gait speed*	–0·213	–0·272, –0·153	< 0·001	–0·076	–0·137, –0·014	0·016	–0·111	–0·172, –0·049	< 0·001	–0·184	–0·243, –0·123	< 0·001
Five-times-sit-to-stand test†	0·131	0·070, 0·192	< 0·001	0·108	0·050, 0·169	0·001	0·164	0·103, 0·223	< 0·001	0·155	0·094, 0·215	< 0·001
SPPB score†	–0·166	–0·225, –0·105	< 0·001	–0·084	–0·145, –0·022	0·008	–0·148	–0·209, –0·087	< 0·001	–0·158	–0·218, –0·097	< 0·001

WWI, weight-adjusted waist index; WC, waist circumference; WHtR, waist-to-height ratio; ASM, appendicular skeletal muscle mass; SPPB, short physical performance battery.

* Pearson’s correlation coefficient.

† Spearman’s correlation coefficient.

The overall correlations between anthropometric indices and physical function measurements were weak; yet, WWI was the only index that showed a significant inverse relationship with physical function measurements in both men and women. Higher WWI was associated with lower maximum grip strength (*r* = −0·245, *P* < 0·001 in men; *r* = −0·215, *P* < 0·001 in women), 4-m usual gait speed (*r* = −0·165, *P* < 0·001 in men; *r* = −0·213, *P* < 0·001 in women) and SPPB score (*r* = −0·138, *P* < 0·001 in men; *r* = −0·166, *P* < 0·001 in women) and a longer time for the five-times sit-to-stand test (*r* = 0·178, *P* < 0·001 in men; *r* = 0·131, *P* < 0·001 in women). Such consistency was not observed in BMI and WC. WHtR showed inverse relationship with physical function measurements, similar to WWI; however, in men, the correlations of WHtR with maximum grip strength (*r* = −0·026, *P* = 0·411) and SPPB score (*r* = −0·048, *P* = 0·124) were not statistically significant.

## Discussion

This is the first study that analysed the association of different anthropometric indices, including BMI, WC, WHtR and WWI, with sarcopenic obesity to test their feasibility as screening tool for sarcopenic obesity. In this study, WWI showed the strongest association with sarcopenic obesity in men and was the best screening tool compared with WHtR, WC and BMI. There was no statistical significance between all four anthropometric indices and sarcopenic obesity in women. Our findings also reported that WWI was the only index that discriminated muscle mass and fat mass in men, while all the anthropometric indices did not in women. Furthermore, WWI was the only index that showed significant inverse association with physical function in both men and women. Taken altogether, WWI has potential to be a simple screening tool for sarcopenic obesity in older men.

In our study, BMI reported similar positive correlations with fat mass and muscle mass in both sexes; we confirmed that BMI’s inability to discriminate fat mass and muscle mass makes it inappropriate to screen sarcopenic obesity. WC is a measure of abdominal obesity that is highly associated with visceral fat^(44,45)^. Although WC showed better association with sarcopenic obesity along with reduced positive correlation with muscle mass compared with BMI, it was still too far to say that WC discriminated muscle mass and fat mass. Nevertheless, BMI and WC were good obesity indicators in line with the previous studies^(46,47)^; WC reported the best correlation with body fat mass in men, while BMI was the best in women. Therefore, we confirmed that BMI and WC cannot solely be used for screening sarcopenic obesity; instead, they have to be combined with physical function indicators^(48,49)^, which may also reflect low muscle mass. WHtR showed significantly better performance as an indicator of sarcopenic obesity in men compared with BMI and WC possibly due its better reflection of body fat distribution by standardising WC for height. However, it was not free from the influence of WC and similar limitations were observed; it still did not discriminate muscle mass and fat mass and was weakly correlated with physical function measurements, especially with muscle strength. Considering the significant association of WHtR and sarcopenic obesity based on all the three criteria in men, WHtR can be a useful indicator in men when combined with appropriate muscle strength indicator, such as grip strength.

In 2021, the WWI was suggested as an indicator that can reflect high fat mass and low muscle mass simultaneously, although the association of WWI with muscle strength and physical performance was not identified^(24)^. Unlike WHtR, WWI standardised WC for weight only and differentiated the effect of height on the same waist^(23)^. In our study, WWI showed a relatively lower correlation with body fat mass compared with the other anthropometric indices for both sexes. However, in men, WWI reflected fat mass and muscle mass in the opposite direction and showed a significant inverse association with physical function. It was the only index that showed a significant positive association with sarcopenia defined as low muscle mass combined with low muscle strength and/or physical performance in men. Consequently, the coexistence of sarcopenia and obesity reported an increased association with the WWI, making it the best index to screen sarcopenic obesity in men compared with the other anthropometric indices. The overall correlations of WWI with muscle mass and each physical function measurement were not strong; still, it was the only index that reflected the components simultaneously while maintaining statistical significance, which led to a better association between WWI and sarcopenic obesity in men compared with the other indices. Meanwhile, WWI did not reflect muscle mass and fat mass in the opposite direction in women as it did in men; it showed the lowest correlation with fat mass compared with the other indices. As a result, WWI was not significantly associated with sarcopenic obesity in women even it showed a significant inverse association with physical function.

The significant sex-specific difference was observed in WWI, WC and WHtR in the association with sarcopenic obesity. Previous studies have shown that men have significantly higher visceral fat and lower extremity fat than women^(50,51)^, while women have higher subcutaneous fat and greater fat infiltration into lower extremity muscles than men^(52,53)^. The sex-specific difference of WWI, WC and WHtR may be attributed to the insufficient reflection of subcutaneous or intermuscular fat. In the previous Health, Aging, and Body Composition Study, a higher amount of subcutaneous fat in women’s lower extremity area was independently associated with slow gait speed^(54)^. We found similar sex-specific difference in mean gait speed in our study population as the proportion of slow gait speed was higher in women. This finding may reflect a higher amount of subcutaneous fat deposited in lower extremity in our women population. In the most recent Multi-Ethnic Study of Atherosclerosis that examined the association between WWI and abdominal fat and muscle mass by CT scans, WWI was not only positively correlated with visceral fat area but also with subcutaneous fat of abdominal area, while negatively correlated with abdominal muscle area^(55)^. Overall, previous findings suggest WC-driven anthropometric indices may not well reflect lower extremity fat that could have interrupted reciprocal assessment of percentage of body fat and ASM/height^2^ in older women in our study. We were not able to confirm this assumption due to the lack of relevant data; further research into the sex-specific differences is needed, especially regarding relationship between the anthropometric indices and distribution of body fat. In addition, a previous study of Korean community-dwelling older adults found that ASM/height^2^ was the most reliable index for sarcopenia in men in terms of predicting functional limitation, while ASM/weight was better in women^(56)^. In light of this, we may need a different approach; applying different muscle indices in our study population could have yielded different results. Although we applied ASM/height^2^ in our study according to the Asian Working Group for Sarcopenia 2019 consensus, studies regarding the application of ASM/weight would be intriguing, and further studies on an anthropometric index for women are warranted.

Our study had some limitations. First, this study included only Korean participants; thus, our findings cannot be generalised to other populations. A multi-ethnic study is required to confirm our findings, especially focusing on physical function. Second, although we calculated the optimal cut-off values of anthropometric indices for the diagnosis of sarcopenic obesity, we could not validate these findings with other scientific evidence as the attempt to apply anthropometric indices to diagnose sarcopenic obesity has not been utilised broadly in the clinical field. It is necessary to establish an appropriate cut-off value for the diagnosis of sarcopenic obesity in clinical practice. Third, the age range of our study was set from 70 to 84 years. We were not able to provide a result for older adults aged 85 years or older; the recruitment of participants with higher ages can be more valuable for this study as the prevalence of sarcopenic obesity increases with age. Finally, this study had a cross-sectional design and causal relationships could not be established; longitudinal studies are required to identify the causal relationship between the anthropometric indices and sarcopenic obesity.

In conclusion, WWI showed the strongest association with three different definitions of sarcopenic obesity and was the best screening tool compared with WHtR, WC and BMI in older men. No anthropometric indices were associated with any definition of sarcopenic obesity in older women. WWI was the only index that was negatively correlated with physical function in both sexes. It has the potential to be utilised as a simple screening tool in clinical practice; however, further research into the sex-specific differences is warranted.

## Supplementary material

For supplementary material accompanying this paper visit https://doi.org/10.1017/S0007114522001817. click here to view supplementary material
